# Effect of Zearalenone on Microbial Spatial Differences in the Chyme and Intestinal Mucosa of Piglets

**DOI:** 10.3390/toxins18040161

**Published:** 2026-03-27

**Authors:** Jiaqi Shi, Kejie Gao, Wenjing Wang, Shengjie Shi, Shuzhen Jiang, Lijie Yang

**Affiliations:** 1 Key Laboratory of Efficient Utilization of Non-Grain Feed Resources (Co-Construction by Ministry and Province), Department of Animal Sciences and Technology, Shandong Agricultural University, Taian 271018, China; sjq77317@outlook.com (J.S.);; 2 Ministry of Agriculture and Rural Affairs, Shandong Provincial Key Laboratory of Animal Nutrition and Efficient Feeding, Department of Animal Sciences and Technology, Shandong Agricultural University, Taian 271018, China

**Keywords:** Zearalenone, weaned piglets, intestinal microbiota, spatial difference, intestinal mucosa, intestinal chyme

## Abstract

Zearalenone (ZEN) is a widely distributed estrogenic mycotoxin that compromises intestinal health in pigs, but its spatial difference ZEN and niche-specific regulatory effects on the intestinal microbiota remain largely unelucidated. In this study, 12 healthy three-way crossbred weaned piglets (Duroc × Landrace × Yorkshire) were randomly divided into two treatments. The control group (CON) was fed with the basal diet, and the treatment group (ZEN) was supplemented with 1.5 mg ZEA/kg of the basal diet for 28 days. Chyme and mucosal microorganisms in the duodenum, jejunum, ileum, colon and cecum were profiled by using 16S rDNA sequencing. The results indicated that ZEN significantly reduced the α-diversity of ileal chyme, while the abnormal increase in α-diversity of ileal and cecal mucosa represented a pathological signature of intestinal mucosal barrier damage induced by ZEN, which was detrimental to intestinal health. β-Diversity analysis revealed ZEN altered the microbial community composition of the cecal chyme. LEfSe analysis revealed gut segment-specific and niche-specific biomarker taxa among the groups, and functional prediction further indicated that ZEN exposure significantly perturbed key metabolic pathways: it downregulated nicotinate and nicotinamide metabolism as well as the citrate cycle in ileal chyme and upregulated the pentose and glucuronate interconversions pathway in cecal chyme. Collectively, this study demonstrated the effects of ZEN on the intestinal microbiota across spatial difference and ecological niches in weaned piglets, providing a basis for elucidating the microecological mechanisms underlying ZEN-induced intestinal injury in pigs.

## 1. Introduction

Zearalenone (ZEN) is an estrogenic mycotoxin that resides in grains and feedstuffs, widely contaminating corn, wheat, barley, and their byproducts [1]. Due to its insensitivity to environmental temperature changes, ZEN is difficult to degrade during food or feed processing and storage, thus posing a persistent threat to global feed safety [2]. Humans may also be exposed to this toxin through the food chain, thus posing a threat to human health [3].

In livestock production, pigs exhibit the highest sensitivity to ZEN among domesticated animals [4]. Pigs typically exhibit reduced fertility, increased embryonic resorption, and decreased litter size after consuming diets containing low levels of ZEN [5,6], while prolonged feeding of high-dose ZEN leads to estrogen excess and mortality in pigs [7]. Among them, weaned piglets are highly vulnerable to ZEN-induced intestinal damage. Despite their basically mature intestinal morphology at this stage, their intestinal mucosal barrier and immune function remain relatively fragile, making them more susceptible to toxin-induced injury [8]. As the primary target organ during ZEN exposure, intestinal damage manifests directly as compromised mucosal barrier integrity, reduced nutrient absorption efficiency, and exacerbated inflammatory responses such as diarrhea, resulting in significant economic losses for the swine industry [9,10]. Previous studies have demonstrated that toxin exposure can alter gut microbial diversity and community structure [11,12]. The series of intestinal injuries induced by ZEN are closely associated with microbial dysbiosis [13,14,15].

Although the impact of ZEN on the porcine gut microbiome has attracted increasing attention, critical knowledge gaps remain. Firstly, most investigations still suffer from sampling bias. Current studies on the porcine gut microbiome predominantly rely on fecal samples or chyme from specific intestinal segments, failing to accurately reflect the dynamic changes in microbiota across different intestinal regions in response to toxins. Secondly, intestinal contents and mucosa represent two distinct functional niches with significant microbial differentiation. The differential sensitivity and response characteristics of microbial communities in these two niches to ZEN exposure remain poorly understood. In swine production, ZEN contamination frequently induces intestinal dysbiosis, diarrhea and growth retardation in weaned piglets, resulting in severe economic losses for pig farms [13]. Elucidating the spatial-specific response patterns of the intestinal microbiota to ZEN exposure can not only provide a scientific basis for developing targeted microbiota regulation strategies but also offer a reference for the clinical early diagnosis of ZEN toxicosis in piglets.

Therefore, this study systematically compared and analyzed the effects of ZEN exposure on chyme and mucosal microbial diversity, community structure, and functional pathways across different intestinal segments in weaned piglets. It clarified the differential responses of microorganisms occupying distinct ecological niches to ZEN. These findings may assist subsequent porcine gut microbiome studies in prioritizing sampling sites while deepening the understanding of ZEN’s differential effects on microorganisms in distinct intestinal niches. This research provides a framework for elucidating the mechanisms underlying ZEN’s gut microecological toxicity.

## 2. Results

### 2.1. The Influence of ZEN on the Spatial Distribution of Intestinal Microbiota

**Figure 1:**
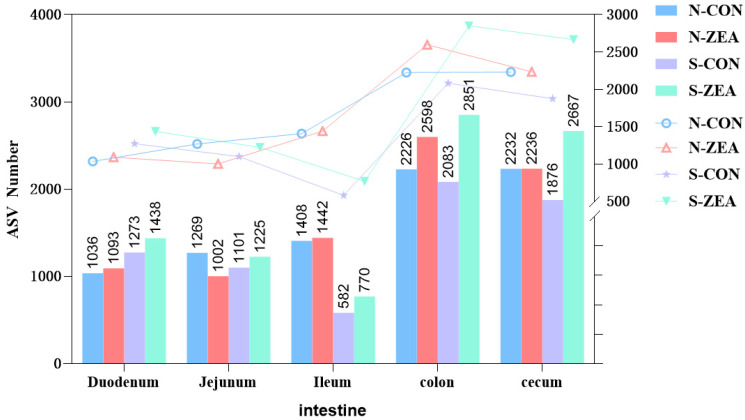
Comparison chart on effect of different treatments on the number of ASVs in chyme and mucosa of each intestinal segment. N-CON represents the mucosal control group, while N-ZEN denotes the mucosal ZEN-treated group, S-CON indicates the chyme control group, and S-ZEN signifies the chyme ZEN-treated group.

To explore the impact of ZEN on the spatial distribution of intestinal microbiota, this study first analyzed the amplicon sequence variants (ASVs) of microbiota in different intestinal segments. Compared to the CON group, ZEN treatment altered the number of ASVs in a segment-specific manner (Figure 1). From a mucosal perspective, ZEN increased the number of ASVs in the colon and cecum. For chyme, the number of ASVs were elevated in the ZEN group across all sites, with more pronounced increases observed in the colonic and cecal chyme. These results indicate that ZEN can have an impact on the spatial distribution of gut microbiota.

**Figure 2:**
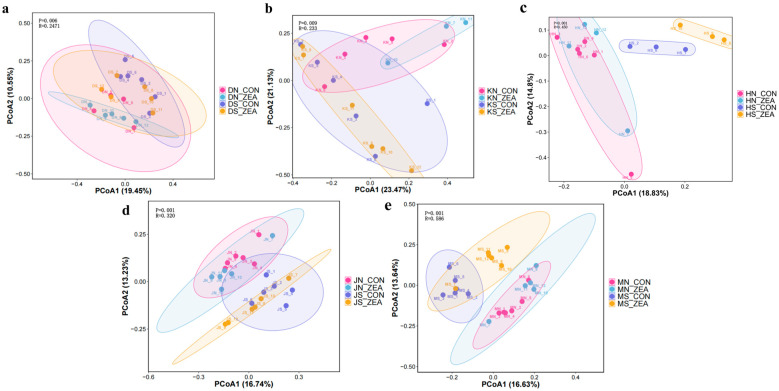
Principal coordinate analysis (PCoA) of β-diversity of mucosal and chyme microbial communities in different intestinal segments. PCoA analyses based on unweighted-unifrac distances are shown in a–e, respectively illustrating microbial community structure differences between mucosa and chyme in different treatment groups of the duodenum (**a**), jejunum (**b**), ileum (**c**), colon (**d**), and cecum (**e**). (DN_CON: Duodenal mucosal control group, DN_ZEA: Duodenal mucosal ZEN-treated group, DS_CON: Duodenal chyme control group, DS_ZEA: Duodenal chyme ZEN-treated group; KN_CON: Jejunum mucosal control group, KN_ZEA: Jejunum mucosal ZEN-treated group, KS_CON: Ileum chyme control group, KS_ZEA: Ileum chyme ZEN-treated group; HN_CON: Ileal mucosal control group, HN_ZEA: Ileal mucosal ZEN-treated group, HS_CON: Ileal chyme control group, HS_ZEA Ileal chyme ZEN-treated group; JN_CON: Colonic mucosal control group, JN_ZEA: Colonic mucosal ZEN-treated group, JS_CON: Colon chyme control group, JS_ZEA: Colon chyme ZEN-treated group; MN_CON: Cecal mucosal control group, MN_ZEA: Cecal mucosal ZEN-treated group, MS_CON: Cecal chyme control group, MS_ZEA: Cecal chyme ZEN-treated group.).

Principal coordinate analysis (PCoA) of β-diversity of mucosal and chyme microbial communities in different intestinal segments revealed significant differences in microbial community β-diversity between chyme and mucosa across all segments, regardless of ZEN treatment (Figure 2). This indicates that chyme and mucosa represent two distinct microhabitats within the piglet intestine, characterized by markedly different microbial composition and structure. ZEN exerts differential effects on microbial communities within these two distinct ecological niches.

### 2.2. ZEN Differentially Alters the α-Diversity of Chyme and Mucosal Microbiota

**Figure 3:**
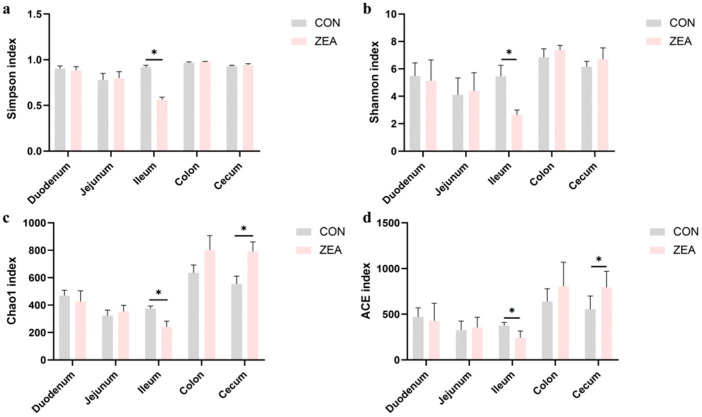
The α-diversity of chyme in each intestinal segment. (**a**–**d**) represent the Simpson index, Shannon index, Chao 1 index, and ACE index of microbial α-diversity in chyme across intestinal segments, respectively. Data are presented as mean ± SD. Independent-samples *t*-test was used for statistical analysis. * *p* < 0.05.

**Figure 4:**
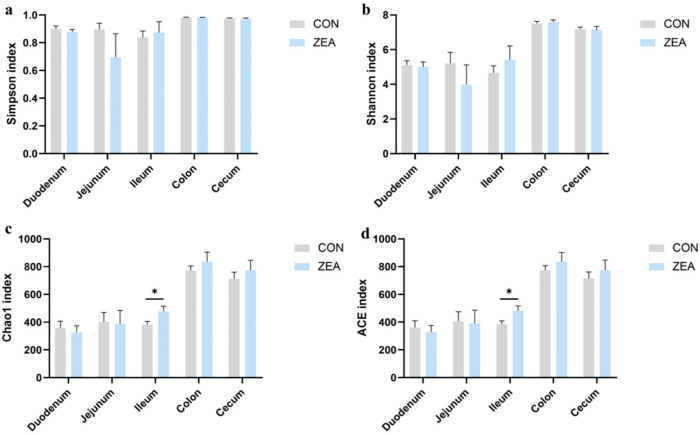
The α-diversity of mucosa in each intestinal segment. (**a**–**d**) represent the Simpson index, Shannon index, Chao 1 index, and ACE index of microbial α-diversity in mucosa across intestinal segments, respectively. Data are presented as mean ± SD. Independent-samples *t*-test was used for statistical analysis. * *p* < 0.05.

To clarify the effects of ZEN on the richness and evenness of gut microbiota in different intestinal segments of piglets, this study conducted α-diversity analyses on chyme and mucosal samples from each intestinal segment. The ZEN-treated group significantly reduced the Simpson index (Figure 3a), Shannon index (Figure 3b), Chao 1 index (Figure 3c), and ACE index (Figure 3d) of ileal chyme while significantly increasing the ACE index in the cecum. The results indicated that the addition of ZEN significantly reduced the richness and uniformity of microorganisms in the ileum chyme and significantly increased the richness of microorganisms in the cecum chyme. For the intestinal mucosa, the ZEN-treated group significantly upregulated the Chao1 index (Figure 4c) and ACE index (Figure 4d) in the ileum but had no effect on other indices (Figure 4). This reveals that the addition of ZEN significantly enhanced the richness of microorganisms in the cecum mucosa.

### 2.3. ZEN Differentially Alters the β-Diversity of Chyme and Mucosal Microbiota

**Figure 5:**
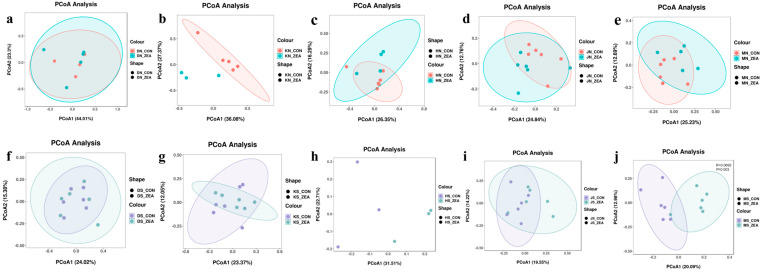
The β-diversity of mucosa and chyme in each intestinal segment. PCoA analyses based on unweighted-unifrac distances (**a**–**j**) reveal microbial community structure differences between mucosa and chyme across various treatment groups in the duodenum (**a**,**f**), jejunum (**b**,**g**), ileum (**c**,**h**), colon (**d**,**i**), and cecum (**e**,**j**). (DN_CON: Duodenal mucosal control group, DN_ZEA: Duodenal mucosal ZEN-treated group, DS_CON: Duodenal chyme control group, DS_ZEA: Duodenal mucosa ZEN-treated group; KN_CON: Jejunum mucosal control group, KN_ZEA: Jejunum mucosal ZEN-treated group, KS_CON: Ileum chyme control group, KS_ZEA: Ileum chyme ZEN-treated group; HN_CON: Ileal mucosal control group, HN_ZEA: Ileal mucosal ZEN-treated group, HS_CON: Ileal chyme control group, HS_ZEA: Ileal chyme ZEN-treated group; JN_CON: Colonic mucosal control group, JN_ZEA: Colonic mucosal ZEN-treated group, JS_CON: Colon chyme control group, JS_ZEA: Colon chyme ZEN-treated group; MN_CON: Cecal mucosal control group, MN_ZEA: Cecal mucosal ZEN-treated group, MS_CON: Cecal chyme control group, MS_ZEA: Cecal chyme ZEN-treated group).

To further analyze the overall impact of ZEN exposure on the gut microbiota structure of piglets and to clarify differences in community composition between the chyme and mucosal niches as well as between intestinal segments, this study performed β-diversity analysis on chyme and mucosal samples from each intestinal segment. ZEN treatment resulted in distinct separation of cecal microbial communities from the control group. (Figure 5j) but had no significant effect on the chyme or mucosa of other intestinal segments (Figure 5a–i). The results indicated that ZEN exposure significantly altered the microbial community structure in the cecum of piglets.

### 2.4. ZEN Alters Microbial Composition at Phylum and Genus Levels in a Site-Dependent Manner

**Figure 6:**
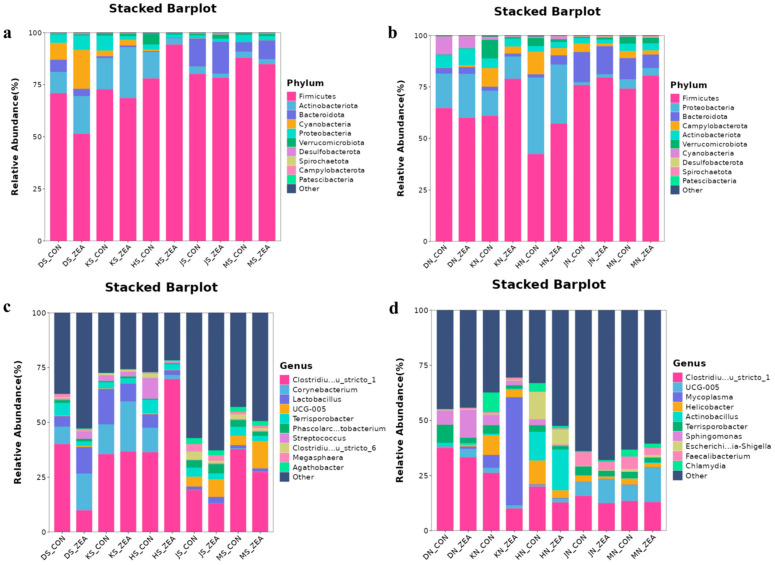
Stacked bar plot showing relative abundance of microbial phylum/genus in ileal and cecal mucosa and chyme samples. (**a**,**c**) display relative abundance of chyme microbial phylum and genus level in respective intestinal segments; (**b**,**d**) display relative abundance of mucosal microbial phyla and genera in respective intestinal segments.

We further analyzed the microbial composition at the phylum and genus levels and performed cluster analysis to evaluate structural differences in the digesta and mucosal microbiota. At the phylum level, *Firmicutes* was the most abundant phylum in both chyme and mucosa (Figure 6a,b). More specifically, in chyme, *Actinobacteriota* were more abundant in the duodenum, jejunum, and ileum, while *Bacteroidota* were more abundant in the colon and cecum (Figure 6a). In mucosa, *Proteobacteria* were the dominant phylum in the duodenum, jejunum, and ileum, whereas *Bacteroidota* were dominant in the colon and cecum (Figure 6b). Similarly, at the genus level, *Clostridium sensu stricto 1* was the most abundant genus in both chyme and mucosa (Figure 6c,d). Comparing control groups with ZEN-treated groups across intestinal segments revealed increased *Corynebacterium* and *Lactobacillus* abundance in duodenal and jejunal chyme contents of ZEN-treated groups. Compared with the control group, dietary ZEN supplementation decreased the relative abundance of *Lactobacillus* by 49.2% in jejunal chyme and by 64.5% in ileal chyme, while *UCG-005* abundance increased in colonic and cecal contents. Comparing the mucosa between the two treatment groups revealed *Mycoplasma* abundance significantly increased in the jejunal mucosa of the ZEN-supplemented group. In the ileal mucosa, the abundance of *Escherichia-Shigella* and *Helicobacter* decreased in the ZEN group, while *Actinobacillus* abundance increased.

### 2.5. Analysis of Differentiation Markers in the Microbial Communities of the Ileum and Cecum Intestinal Mucosa and Chyme

**Figure 7:**
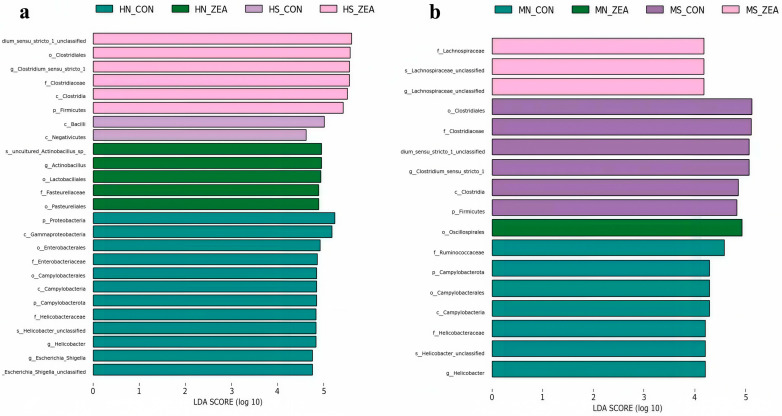
LEfSe analysis of differential microbial taxa in ileal (**a**) and cecal (**b**) chyme and mucosa samples (LDA score > 4.0). (HN_CON: Ileal mucosal control group, HN_ZEA: Ileal mucosal ZEN-treated group, HS_CON: Ileal chyme control group, HS_ZEA: Ileal chyme ZEN-treated group; MN_CON: Cecal mucosal control group, MN_ZEA: Cecal mucosal ZEN-treated group, MS_CON: Cecal chyme control group, MS_ZEA: Cecal chyme ZEN-treated group).

Based on the results of the α and β-diversity analyses described earlier, ZEN exhibits significant segment-specific disruption of the intestinal microbiota in piglets. Among the intestinal segments, the ileum and cecum showed the most pronounced changes in microbial diversity and community structure following ZEN exposure. Therefore, this study further analyzed the microbial communities in the ileum and cecum using LEfSe analysis, with the results presented in Figure 7.

In the ileal mucosa, the characteristic microbiota of the control group primarily comprised *Proteobacteria*, *Enterobacterales*, *Campylobacterota*, *Helicobacteraceae*, and *Escherichia_Shigella*. The ZEN group’s characteristic microbiota primarily comprised uncultured_*Actinobacillus_sp*, *Actinobacillus*, *Lactobacillales*, and *Pasteurellaceae*. In ileal chyme, the control group’s characteristic microbiota mainly consisted of Bacilli and Negativicutes, while the ZEN group’s characteristic microbiota was dominated by *Clostridiales* and *Firmicutes* (Figure 7a). In the cecal mucosa, the characteristic microbiota of the control group primarily comprised *Ruminococcaceae*, *Campylobacterota*, and *Helicobacteraceae*; the ZEN group’s characteristic microbiota was dominated by *Oscillospirales*. In cecal chyme, the characteristic microbiota of the control group primarily comprised *Clostridiales*; the ZEN group’s characteristic microbiota was dominated by *Lachnospiraceae* (Figure 7b). Results indicate that ZEN exerts niche- and group-specific effects on microbial communities in the ileum and cecum of piglets.

### 2.6. Functional Predictive Analysis of the Microbial Communities in the Ileum and Cecum Mucosa and Chyme

**Figure 8:**
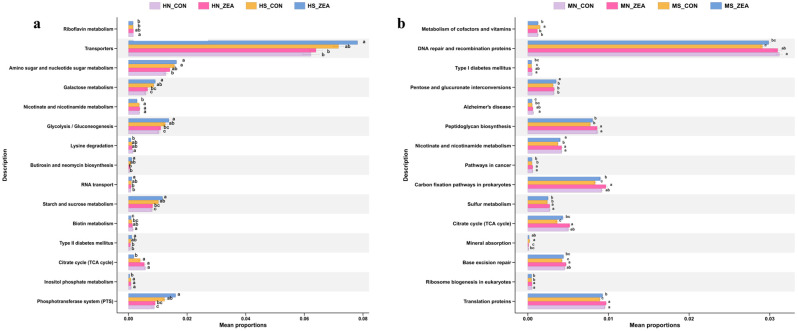
Relative abundance distribution of KEGG level 3 functional pathways in ileal and cecal digesta and mucosa samples. (**a**,**b**) Functional potential prediction of the ileal, cecal mucosal, and chyme microbial communities using PICRUSt2 version 2.6.2 software, annotated to KEGG level 3 functional pathways, showing the relative abundance distribution of microbial functional pathways across different treatment groups. (**a**) represents PICRUSt2-based KEGG functional prediction (level 3) for ileal mucosal and chyme microbial communities; (**b**) shows PICRUSt2-based KEGG functional prediction (level 3) for cecal mucosa and chyme microbiota. (HN_CON: Ileal mucosal control group, HN_ZEA: Ileal mucosal ZEN-treated group, HS_CON: Ileal chyme control group, HS_ZEA: Ileal chyme ZEN-treated group; MN_CON: Cecal mucosa control group, MN_ZEA: Cecal mucosa ZEN-treated group, MS_CON: Cecal chyme control group, MS_ZEA: Cecal chyme ZEN-treated group. Lowercase letters a, b, c on the same bar denote statistically significant differences between groups (*p* < 0.05), while identical letters indicate no significant differences).

To investigate the effects of ZEN exposure on the functional potential of intestinal microbiota in piglets, the top 15 pathways with the smallest *p*-values at level 3 were selected based on 16S rRNA sequencing data. Functional annotation of microbial communities was performed using PICRUSt2. Overall, the functional potential of gut microbiota across the four groups primarily concentrated in metabolic pathways, disease-related pathways, genetic information processing pathways, and nutrient absorption pathways, as shown in Figure 8.

In the ileum (Figure 8a), the abundance of Transporters was significantly higher in both chyme and mucosal samples compared to other pathways. Among material metabolism pathways, the relative abundance of the amino sugar and nucleotide sugar metabolism pathway was significantly higher in the mucosal ZEN group than in the control group. In chyme samples (Figure 8b), the abundance of Nicotinate and nicotinamide metabolism, citrate cycle, and Inositol phosphate metabolism pathways was significantly reduced in the ZEN group (*p* < 0.05), while no significant differences were observed among other metabolic pathways. In the cecum, the abundance of DNA repair and recombination proteins was significantly higher in both chyme and mucosal samples than in other pathways. Among material metabolism pathways, the relative abundance of Pentose and glucuronate interconversions and Nicotinate and nicotinamide metabolism pathways was significantly higher in the ZEN group than in the control group in chyme; conversely, the relative abundance of metabolism of cofactors and vitamin pathway was significantly higher in the chyme control group than in the ZEN group. No significant differences were observed among other metabolic pathways.

## 3. Discussion

This study revealed the spatial differences in microbial communities within the chyme and intestinal mucosa of different intestinal segments in piglets exposed to ZEN through 16S rRNA sequencing. Our principal finding is that ZEN exposure induces segment-specific and niche-specific dysbiosis. The most profound alterations were observed in the ileum and cecum, rather than in the duodenum and jejunum where ZEN primarily accumulates. This dysbiosis was characterized by a decline in beneficial commensals, a shift in key bacterial populations, and distinct diversity patterns between the chyme and mucosa, suggesting multifaceted mechanisms of ZEN toxicity on gut ecosystem stability and function [16].

Previous studies have indicated that the duodenum and jejunum are the primary sites of ZEN absorption [17]. However, this study found that the sites of ZEN-induced disruption to gut microbiota do not coincide with the sites of toxin accumulation. This study reveals that the ileum is the primary intestinal segment disrupted by ZEN, manifesting as reduced microbial diversity in chyme and increased diversity in the mucosa—an inverse outcome potentially linked to the ileum’s physiological characteristics. The ileum serves as the terminal absorption site for vitamin B_12_, bile acids, and carbohydrates and amino acids not absorbed in the jejunum, while also constituting the primary region for the intestinal mucosal immune barrier [18,19,20]. Weaned piglets exhibit particular sensitivity to ZEN, with even low doses causing adverse health effects [21]. Previous studies have shown that low-dose ZEN only slightly affects the colonic microbial composition of weaned piglets, whereas high-dose ZEN induces disturbances in the gut microbiota structure. Collectively, these results demonstrate that the effects of ZEN on the gut microbiota of piglets are dose-dependent [21,22]. As the primary absorption site, the ileum accumulates locally high concentrations, making it a focal region for ZEN toxicity. Research by Wang [23] on mouse intestinal mucosal microbiota revealed that ZEN exposure severely disrupted the structural integrity of the jejunal mucosa and altered the balance of the gut microbiota. The compromised integrity of the intestinal mucosal barrier led to an imbalance in the intestinal environment, creating opportunities for opportunistic pathogens to colonize and proliferate [24]. Intestinal barrier function is mainly dependent on tight junctions, which are crucial for forming selectively permeable seals between adjacent intestinal epithelial cells [25]. Tight junctions are primarily composed of transmembrane proteins, tight junction-associated proteins, junctional adhesion molecules, and cytoplasmic proteins. ZEN can disrupt these tight junction structures, increase intestinal permeability, and further impair intestinal barrier integrity [26]. This finding is consistent with the increase in α-diversity of the ileal mucosal microbiota observed in this study. *Lactobacillus* is one of the key probiotic genera in the gut, playing a vital role in maintaining intestinal health [27]. However, ZEN exerts an inhibitory effect on probiotics. Research by Liu [28] demonstrated that *Lactobacillus* abundance was significantly reduced in sows fed ZEN, and levels of *Lactobacillus* and *Bifidobacterium* remained diminished at 21 days post-farrowing. This further supports the findings in this study showing significantly reduced richness and evenness of ileal chyme microbiota. ZEN affects the digestive and absorptive functions of the small intestine. Disaccharidases are key enzymes for carbohydrate digestion and absorption in the small intestine, and their reduced activity directly leads to increased levels of undigested carbohydrates in food [29]. Research by Liu et al. [30] indicates that ZEN impacts disaccharidase activity in the small intestines of weaned gilts, reducing their carbohydrate digestibility. Unabsorbed carbohydrates entering the cecum provide a rich nutrient substrate for the cecal microbiota, leading to increased abundance of cecal chylomicron-associated microbiota, consistent with the findings of this study. The core regulatory mechanism underlying intestinal dysbiosis induced by ZEN is conserved among monogastric animals. However, weaned piglets possess a unique intestinal physiological state that differs from adult sows and mice, resulting in distinct sensitivity and response patterns of the gut microbiota to ZEN exposure. Research indicates that ZEN suppresses colitis by promoting the growth of short-chain fatty acid-producing bacteria [31]. Bacillus väthii strain A2 alleviates ZEN-induced cecal inflammation by regulating the gut microbiota and short-chain fatty acid levels [32,33]. These findings collectively support the sensitivity of cecal microbial communities to substrate changes and suggest ZEN may indirectly alter cecal microbial abundance by influencing carbohydrate absorption.

Genus-level analysis revealed a significant decrease in *Lactobacillus* abundance in ileal chyme. *Lactobacillus* is a typical probiotic that produces short-chain fatty acids to maintain gut health [34]. Previous studies have shown that ZEN can influence the gut microbiota by reducing the abundance of specific strains, including *Lactobacillus* [35]. Conversely, an increase in Actinomyces abundance was observed in the ileal mucosa, suggesting that ZEN induces a pro-inflammatory shift in the intestinal environment, and multiple prior studies have demonstrated this conclusion [36,37]. *Helicobacter* is a potentially pathogenic intestinal commensal bacterium that can disrupt the intestinal barrier function in pigs and induce low-grade inflammation [38]. Notably, the abundance of *Escherichia-Shigella* and *Helicobacter pylori* in the mucosa decreased. This finding appears to contradict the common conclusion that ZEN increases the abundance of pathogenic bacteria, but it may be related to competitive interactions among microorganisms [39,40]. When microorganisms are exposed to ZEN, *Actinobacteria* or other potentially ZEN-tolerant microbial communities proliferate rapidly. By competing for attachment sites and nutrients on the mucosa, they crowd out the survival space of other pathogens. *Clostridium sensu stricto 1* constitutes a core bacterial community in piglet intestinal chyme and belongs to strictly anaerobic bacteria. Certain strains within this genus possess the ability to produce butyrate [41]. A decrease in their abundance may lead to reduced local butyrate levels in the cecum, potentially resulting in intestinal barrier dysfunction [42].This aligns with the findings of this study indicating a significant enrichment of functional pathways related to glucose metabolism in ileal chyme.

## 4. Conclusions

This study investigated the spatial effects of 1.5 mg ZEN/kg of the basal diet on the microbiota in chyme and intestinal mucosa across different intestinal segments of weaned piglets using 16S rDNA sequencing and clarified that ZEN exerts segment-specific and niche-specific regulatory effects on the intestinal microbiota of piglets. These findings provide an experimental basis for elucidating the microecological mechanism of ZEN-induced intestinal injury in piglets and lay a theoretical foundation for developing targeted prevention and control strategies against ZEN toxicosis in piglets. This study has certain limitations: the contents of ZEN and its metabolites in different intestinal segments were not determined, failing to directly establish the correlation between the local ZEN exposure level and microbial community changes. In future research, the detection of ZEN and its metabolite contents in different intestinal segments can be incorporated to clarify the dose–effect relationship between ZEN exposure and intestinal microbial dysbiosis. Meanwhile, probiotics that can reverse microbial dysbiosis in the ileum and cecum can be screened and combined with the detection of indicators such as intestinal tight junction proteins and inflammatory factors to provide more precise technical support for the prevention and control of ZEN toxicosis in piglets.

## 5. Materials and Methods

### 5.1. Preparation of Zearalenone (ZEN) and Experimental Diets

ZEN was purchased from Fermentek Ltd. (Jerusalem, Israel) and was of chromatography-grade purity, with a certified purity of ≥98%. Zearalenone (ZEN) was fully dissolved in ethyl acetate via shaking, and the solution was evenly sprayed onto a quantified amount of talcum powder. The mixture was blended thoroughly using a shaker to prepare a ZEN premix at a concentration of 1000 mg/kg. The premix was placed in a fume hood at room temperature for 24 h to allow complete evaporation of ethyl acetate. The prepared 1000 mg/kg ZEN premix was then serially diluted with an appropriate amount of basal diet to obtain the experimental diet containing 1.5 mg/kg ZEN. All experimental diets were stored sealed in a cool, dry, and well-ventilated area. According to the Method for Sampling Feed (GB/T 14699.1-2005) [43], samples of the experimental diets were collected at the beginning and the end of the experiment, respectively, for the analysis of proximate nutrients and mycotoxin concentrations.

### 5.2. Animal Experiment Design

In this study, a total of twelve healthy (Duroc × Landrace × Yorkshire) crossbred weaned piglets were selected at 28 days of age (IACUC:SDAUA-2025-138). A 28-day duration is sufficient to induce consistent and stable changes in intestinal microbiota, growth performance, and intestinal health without causing excessive stress or acute mortality [44]. Piglets were transferred to the experimental facility at 42 days of age (approximately 6 weeks old) and allowed a 2-day acclimation period. Subsequently, they were randomly assigned to two dietary treatments with six pigs per treatment. Initial body weight did not differ significantly between treatment groups (*p* > 0.05). The control group received a basal diet formulated to meet the nutrient requirements of weaned piglets according to NRC (2012) [45] guidelines. The experimental group received the same basal diet supplemented with 1.5 mg/kg ZEN. The composition and nutritional profile of the basal diet are presented in Table 1.

### 5.3. Husbandry and Management

The animal experiment was conducted at the Experimental Station of the College of Animal Science and Technology, Shandong Agricultural University, from April to June 2024. All piglets were housed in metabolic cages equipped with nipple drinkers, feed troughs, and slatted floors to ensure ad libitum access to feed and water. Before the initiation of the experiment, all facilities and equipment in the pig house were carefully inspected, followed by thorough cleaning and disinfection of both the interior and exterior of the pig house. During the experimental period, the exterior of the pig house was disinfected daily, and the interior was disinfected weekly. Adequate ventilation was maintained in the pig house throughout the day, and the pig house was cleaned regularly in the morning, noon, and evening. The ambient temperature was set at 30 °C during the first week of the experiment and maintained at 25–28 °C from the second week onward, with the relative humidity kept at approximately 65%. All other husbandry practices were performed according to standard procedures until the end of the experiment. All piglets were slaughtered upon completion of the experiment. The health status of piglets was closely monitored, and routine immunization procedures were implemented throughout the experiment.

### 5.4. Determination of Dietary Nutrient and Mycotoxin Contents

Proximate nutrients in the diets were determined according to AOAC (2012) [46]. Feed samples were dried to constant weight in a 65 °C oven, ground, and passed through a 40-mesh sieve. Dry matter (DM) content was determined by drying at (103 ± 2) °C (GB/T 6435-2014) [47]. Crude ash (CA) was analyzed using the 550 °C ashing method (GB/T 6432-2018) [48]. Acid-insoluble ash (AIA) was determined using the endogenous indicator method (GB/T 23742-2009) [49]. Organic matter (OM) was calculated as follows: Organic matter (OM) = Dry matter (DM) − Crude ash (CA). Crude protein (CP) was measured using the semi-micro Kjeldahl method (GB/T 6432-2018). Ether extract (EE) was determined by the Soxhlet ether extraction method (GB/T 6433-2006) [50].

Deoxynivalenol (DON), aflatoxin B1 (AFB1), fumonisin (FUM), and zearalenone (ZEA) were quantitatively determined by immunoaffinity column purification coupled with high-performance liquid chromatography (HPLC). The limits of detection (LOD) for DON, AFB_1_, FUM, and ZEA were 0.1 mg/kg, 0.01 mg/kg, 0.25 mg/kg, and 0.1 mg/kg, respectively.

### 5.5. Growth Performance

Piglets were weighed individually at the beginning and the end of the formal experiment. Daily feed intake and residual feed were recorded for each piglet. Average daily feed intake (ADFI), average daily gain (ADG), and feed-to-gain ratio (F/G) were calculated as follows:
Average daily feed intake (ADFI, kg/d) = Total feed offered − Residual feedAverage daily gain (ADG, kg/d) = (Final body weight − Initial body weight)/Experimental daysFeed-to-gain ratio(F/G) = Average daily feed intake (kg/d)/Average daily gain (kg/d)

### 5.6. Sampling Methods

#### 5.6.1. Collection of Intestinal Contents

Following the conclusion of animal testing, all piglets were euthanized. All surgical instruments used in the experiment were sterilized beforehand by soaking in 75% ethanol. After euthanasia, the muscles medial to the scapula and surrounding the hip joint were transected transversely. The limbs were abducted laterally, leaving only the skin connected to the trunk, positioning the carcass in a supine position. An abdominal incision was then made along the midline of the abdominal wall from posterior to the xiphoid process to the symphysis pubis, extending simultaneously from the xiphoid process along the posterior margins of the ribs toward the transverse processes of the lumbar vertebrae. The abdominal wall was divided into two symmetrical wedge-shaped skin flaps and retracted laterally. The small intestine was shifted to the left to expose the rectum. After a single ligation of the rectum within the pelvic cavity, it was transected. The left hand grasped the proximal rectum while the right hand held a scalpel to incise the mesenteric root and surrounding connective tissue from the rectum toward the diaphragm. Upon reaching the diaphragm, the esophagus was singly ligated and transected anterior to the cardia. The entire intestinal tract was then completely dissected. We segmented the dissected digestive tract into the duodenum, jejunum, ileum, colon, and cecum. Both ends of each segment were ligated before separating them individually. We placed each isolated digestive segment on sterile aluminum foil. Using sterile medical scissors, small incisions were made in the middle of the stomach and each intestinal segment. With a sterile curette, approximately 2 g of intestinal contents were collected. We divided the contents into sterile 2 mL cryovials, tightly screwed on the caps, and immediately stored in liquid nitrogen and subsequently transferred to −80 °C until analysis. The interval between clinical death and sample freezing was 20 min.

#### 5.6.2. Collection of Porcine Gastrointestinal Mucosa

Each separated digestive segment was placed on sterile tin foil, using 10 cm segments from each of the intestinal regions. The inner wall of the excised tissues was gently rinsed with sterile normal saline until no visible contents remained adhered to the surface. Each intestinal segment was then longitudinally incised along the midline, and the intestinal mucosa was gently scraped using a sterile glass slide at an angle of approximately 45° relative to the tissue surface. The scraped mucosa was aliquoted into sterile 2 mL cryovials, temporarily stored in liquid nitrogen and subsequently transferred to −80 °C until analysis. The interval between clinical death and sample freezing was 20 min.

### 5.7. 16S rDNA Gene Fragment Amplification and Sequencing

Genomic DNA was extracted from intestinal chyme and mucosa samples using the E.Z.N.A.Stool DNA Kit (Omega Bio-tek, Norcross, GA, USA) according to the manufacturer’s protocol. The microbiota of the control group at the start of the experiment was regarded as the baseline microbiota for comparison. DNA quality was verified by 1% agarose gel electrophoresis, and concentration was quantified using a UV spectrophotometer (NanoDrop, Thermo Fisher Scientific, Wilmington, DE, USA). The V3–V4 hyper variable region of the bacterial 16S rRNA gene was amplified with barcoded primers 341F (5′-CCTACGGGNGGCWGCAG-3′) and 805R (5′-GACTACHVGGGTATCTAATCC-3′). PCR products were confirmed by 2% agarose gel electrophoresis, purified with AMPure XT beads (Beckman Coulter Genomics, Danvers, MA, USA), and quantified using a Qubit Fluorometer (Invitrogen, Carlsbad, CA, USA). Amplified libraries were pooled in equimolar ratios. Library size distribution was assessed on an Agilent 2100 Bioanalyzer (Agilent Technologies, Santa Clara, CA, USA), and quantification was performed using the Kapa Library Quantification Kit (Kapa Biosystems, Wilmington, MA, USA). Paired-end sequencing (2250 bp) was conducted on an Illumina NovaSeq 6000 platform (LC-Bio, Hangzhou, Zhejiang, China).

### 5.8. Process of Sequencing Data

Raw paired-end sequences were demultiplexed based on sample-specific barcodes, followed by removal of adapter and primer sequences. Reads were merged using FLASH v1.2.11. Quality filtering was performed with fqtrim v0.94 to generate high-quality clean tags. Chimeric sequences were removed with Vsearch v2.3.4. Denoising and generation of amplicon sequence variants (ASVs) were conducted using DADA2 in QIIME2.

### 5.9. Statistical Analysis

Alpha diversity (Chao1, ACE, Shannon, Simpson indices) was calculated in QIIME2 after rarefaction to the minimum sequencing depth. Results were visualized with GraphPad Prism v8.0. Independent-samples *t*-test was used for statistical analysis.

Beta diversity was assessed using Bray–Curtis dissimilarity and visualized via principal coordinate analysis (PCoA) in R v3.5.2 (phyloseq package). Taxonomic assignment of representative sequences was performed against the SILVA database (release 132) using BLAST 2.12.0 with 97% similarity threshold.

## Figures and Tables

**Table 1:** Ingredients and nutrient contents of the basal diet (%, air-dry basis).

Ingredients	Content (%)	Nutrients	Analyzed Values
Expanded corn	64.43	Metabolizable energy, MJ/Kg	13.86
Whey powder, CP 3%	5.00	Crude protein, mg/kg	18.48
Fermented soybean meal	14.00	Calcium, mg/kg	0.74
Expanded soybean	8.50	Total phosphorus, mg/kg	0.62
Fish meal, CP 63.28%	4.00	STTD phosphorus, mg/kg	0.41
CaHPO_4_	1.15	ATTD phosphorus, mg/kg	0.38
Pulverized limestone	0.70	Lysine, mg/kg	1.38
NaCl	0.20	Methionine, mg/kg	0.40
L-Lysine HCl	0.76	Sulfur amino acid, mg/kg	0.66
DL-Methionine	0.08	Threonine, mg/kg	0.85
L-Threonine	0.16	Tryptophan, mg/kg	0.23
L-Tryptophan	0.02		
Premix	1.00		
Total	100.00		

Note. Supplied per kg of diet: vitamin A, 3300 IU; vitamin D_3_, 330 IU; vitamin E, 24 IU; vitamin K_3_, 0.75 mg; vitamin B_1_, 1.50 mg; vitamin B_2_, 5.25 mg, vitamin B_6_, 2.25mg; vitamin B_2_, 0.026 mg, pantothenic acid, 15.00 mg; niacin, 22.50 mg; biotin, 0.075 mg; folic acid, 0.45 mg; Mn (MnSO_4_·H_2_O), 4.00 mg; Fe (FeSO_4_·H_2_O), 90 mg; Zn(ZnS0_4_·H_2_0), 90 mg; Cu (CuSO_4_·5H_2_0),6.00 mg; I (KIO_3_), 0.14 mg, Se (Na_2_SeO3), 0.30 mg.

## Data Availability

The raw data supporting the conclusions of this article will be made available by the authors on request.
